# Commentary: Respiration-Entrained Brain Rhythms Are Global but Often Overlooked

**DOI:** 10.3389/fnsys.2018.00025

**Published:** 2018-06-05

**Authors:** Andrew W. Corcoran, Giovanni Pezzulo, Jakob Hohwy

**Affiliations:** ^1^Cognition and Philosophy Laboratory, School of Philosophical, Historical and International Studies, Monash University, Melbourne, VIC, Australia; ^2^Institute of Cognitive Sciences and Technologies, National Research Council, Rome, Italy

**Keywords:** respiration, neural oscillation, active sensing, olfaction, active inference, free energy principle, prediction, interoceptive rhythm

Tort et al. (2018a) provide a timely review of the growing body of evidence implicating respiration-entrained oscillations as a distinctive class of rhythmic brain activity. In rodents, these olfactory-driven “respiratory rhythms” are dissociable from concomitant low-frequency oscillations in the delta and theta bands, and have been shown to modulate high-frequency (gamma) activity in non-olfactory regions. Tort et al. (2018a; see also Zhong et al., 2017) hypothesize that these rhythms help co-ordinate the integration of distributed neural assemblies, echoing recent claims that breathing-related afferent input constitutes a fundamental organizing principle of oscillatory brain activity (Heck et al., 2017; Herrero et al., 2018). They stop short however of speculating about the precise functional role of these dynamics within the scheme of cognitive processing.

Certainly, more work needs to be done to establish the pervasiveness of respiratory rhythms with respect to non-olfactory processing, and to rule out potential confounds such as volume conduction (see Tort et al., 2018b). This caveat notwithstanding, we propose an elaboration of Tort et al.'s (2018a) hypothesis that interprets respiration-entrained oscillations in the context of *active sensing*, before briefly considering how this account might be subsumed under the *active inference framework*.

## 1. Active sensing and temporal prediction

Active sensing captures the idea that biological sensation is typically contingent upon selective sampling routines involving motor and attentional processes (Schroeder et al., 2010). The dependence of mammalian olfaction on the induction of airflow through the nasal cavity, and the ease with which respiration can be modulated to optimize odor perception (Verhagen et al., 2007), make it a paradigmatic example of active sensing (Wachowiak, 2011).

In freely-exploring rodents, sniffing bouts are systematically coupled with rhythmic nasal, head, and whisking movements, resulting in complex ensembles of phase-locked behavior (Welker, 1964; Deschênes et al., 2012; Moore et al., 2013; Ranade et al., 2013). As previously suggested (see for e.g., Buonviso et al., 2003; Kepecs et al., 2006; Kleinfeld et al., 2014; Kurnikova et al., 2017), one advantage of this arrangement is that multiple sensorimotor channels can be co-ordinated to optimize the selective integration (or segregation) of various streams of information. Active sensing implies that the rhythmically-patterned activity induced by sniffing and whisking establishes a temporal regime under which discrete bursts of afferent input coincide with phasic cycles of neuronal excitability. This process is posited to enhance the gain on relevant sensory information, effectively amplifying (or filtering) salient signals at the expense of competing or irrelevant stimuli (Schroeder et al., 2010; Morillon et al., 2015). From a predictive processing perspective, rhythmic activity induced by sniffing and whisking may specifically support *predictive timing* and the necessary temporal alignment of bottom-up sensory streams with top-down predictive streams (Arnal and Giraud, 2012; Pezzulo et al., 2017).

## 2. Respiration as active sensing

We believe that framing respiration-entrained oscillations in terms of active sensing and temporal prediction delivers at least three useful insights.

First, this perspective contextualizes respiratory rhythms within a more general account of oscillatory-driven attentional selection and sensory enhancement (Lakatos et al., 2008; Schroeder and Lakatos, 2009). This scheme is of course entirely consistent with Tort et al.'s (2018a) hypothesis that respiratory rhythms facilitate inter-regional communication via cross-frequency coupling. Furthermore, it also accommodates complementary behavioral evidence that non-olfactory sensorimotor processing is modulated by respiratory phase (Schulz et al., 2016), thus yielding a framework that may unify these lines of research.

Second, the intrinsically cyclical nature of the sensorimotor dynamics assumed under active sensation encourages us to conceive of respiratory rhythms as being coherent with (or even the consequence of) central processes—namely, *endogenous* brain oscillations—rather than in terms of purely bottom-up sensory entrainment. That is, even though the activity of the neural circuits controlling respiration may not be sufficient to induce respiration-entrained oscillations *per se* (Tort et al., 2018a), these generators are ultimately responsible for dictating the precise timing of nasal stimulation, and thus for promoting the temporal co-ordination between internal brain dynamics and the active sampling of sensory stimuli. This remark highlights a subtle distinction between neural entrainment to (centrally-driven) respiratory cycles, vs. more commonly-described forms of entrainment to (passively received) external stimuli.

Relatedly, we note that the central co-ordination of respiratory timing likewise distinguishes breathing-related (re)afferent input from the periodic interoceptive signals generated by autonomous peripheral pacemakers, such as the heart and gut. This raises the question of whether the reafferent feedback loops established by respiration are in some sense unique, or whether other sources of rhythmically-patterned autonomic activity might similarly contribute to the organization of neural oscillations. Indeed, given that the cardiac cycle is known to influence sensory and cognitive processing (Critchley and Garfinkel, 2018), is itself modulated by respiratory phase (Berntson et al., 1993), and correlates with sniffing/whisking-entrained limbic oscillations in rats (Komisaruk, 1970), future research might profit from delineating the intricate oscillatory dynamics structuring neuro-cardio-respiratory interactions.

## 3. Respiration as active inference

Finally, active sensing implies the instantiation of spatiotemporal predictions about the existence of relevant sensory objects in the external world (Morillon et al., 2015). We believe this account is compatible with (and indeed, subsumed by) active inference, a biologically-plausible implementation of the *free energy principle* (Friston et al., 2017a). Under this scheme, perception and action serve to minimize *variational free energy*, an information-theoretic bound on the *surprise* (negative log-probability) induced by sensory states, given a probabilistic (generative) model of the causes of those states (Friston, 2010). While perception minimizes the difference between current and expected states to infer the most probable causes of sensory inputs, action seeks new sensory inputs that realize expected (i.e., goal) states. In other words, agents are most likely to select actions (and action sequences or *policies*) that minimize the *expected free energy* of future states (Friston et al., 2015).

A corollary of this formulation is that action serves to optimize the trade-off between *epistemic value* (i.e., information gain) and *expected utility* (i.e., reward). On this view, sniffing/whisking cycles can be construed as instances of “epistemic foraging,” where the rodent acts to reduce uncertainty about the state of their environment by engaging in behaviors that disclose its structure and affordances (Friston et al., 2015, 2017a,b). (Note that bouts of active sensory sampling may be embedded within larger-scale periodic activities, such as repetitive patterns of spatial navigation; see Lebedev et al., 2018). Once ambiguity or uncertainty has been sufficiently resolved, the rodent may then switch to policies that exploit the resources availed by the environment. This perspective thus situates active sensation within the broader contexts of predictive processing, information gain and policy selection, while also relating respiratory-driven oscillatory dynamics to more general imperatives governing physiological regulation—as in active inference, uncertainty-reduction is ultimately functional to selecting adaptive action (Pezzulo et al., 2015, 2018).

## 4. A global scheme of hierarchically-nested rhythms?

Beyond the rodent literature, Tort et al. (2018a) briefly consider emerging evidence that nasal-respiration exerts analogous influences over non-olfactory cortical oscillations in humans. Since human respiratory rates typically fall below the delta band (<0.5 Hz), these findings support the notion that the low-frequency rhythms typically studied in M/EEG experiments might themselves be nested within slow (and perhaps in turn, ultra-slow) oscillations (cf. Penttonen and Buzsáki, 2003; Klimesch, 2013). Extending this idea, we suggest that additional, interoceptive sources of slow oscillatory input (e.g., baroreceptor feedback) might likewise constitute global (but often overlooked) rhythms structuring higher-frequency brain activity (see Richter et al., 2017; Mather and Thayer, 2018; Rebollo et al., 2018, for evidence corroborating this view). This picture invites consideration of the inherent causal circularity at the heart of brain-body interactions, whereby endogenous neural oscillations and rhythmic physiological fluctuations reciprocally condition and constrain one another. Active inference, insofar as it encompasses hierarchical temporal predictions integrating across autonomic and somato-sensorimotor loops, presents an ideal framework for generating and testing such hypotheses.

## Author contributions

AC drafted the first version of the manuscript. All authors contributed to manuscript revision, read, and approved the submitted version.

### Conflict of interest statement

The authors declare that the research was conducted in the absence of any commercial or financial relationships that could be construed as a potential conflict of interest.
